# Prognosis and Risk Factors of Recurrence in HBV-Related Small Hepatocellular Carcinoma After Stereotactic Body Radiation Therapy

**DOI:** 10.3389/fonc.2022.903355

**Published:** 2022-07-25

**Authors:** Yifan Han, Jianxiang Liu, Jiali Pan, Hongyu Chen, Ning Tan, Qian Kang, Yuqing Yang, Xiaoyuan Xu, Wengang Li

**Affiliations:** ^1^ Department of Infectious Diseases, Peking University First Hospital, Peking University, Beijing, China; ^2^ Department of Gastroenterology, Peking University First Hospital, Peking University, Beijing, China; ^3^ Radiation Oncology Department, Fifth Medical Center of Chinese PLA General Hospital, Beijing, China

**Keywords:** hepatocellular carcinoma, stereotactic body radiation therapy, hepatitis B virus, aspartate aminotransferase-to-platelet ratio index, recurrence

## Abstract

**Objective:**

The role of stereotactic body radiation therapy (SBRT) for treating small hepatocellular carcinoma (sHCC) has gained increasing recognition. However, the prognosis and risk factors for recurrence in patients with sHCC remain unclear. This study investigated the risk factors for the recurrence of hepatitis B virus (HBV)-related sHCC after SBRT.

**Methods:**

A total of 240 HBV-related sHCC patients treated with SBRT between March 2011 and March 2020 were retrospectively analyzed. The cumulative probability of recurrence was calculated according to the Kaplan–Meier method. Univariate and multivariate analyses were performed with Cox proportional hazard models.

**Results:**

Recurrent hepatocellular carcinoma developed in 134 (55.8%) patients at a median time of 27 months after SBRT. The one- and two-year rates of recurrence were 20.9 and 45.0%, respectively. The median follow-up time was 30 months. The Cox multivariate analysis indicated that age (P = 0.029, HR [1.019, 1.002–1.037]), tumor size (P = 0.012, HR [1.227, 1.045–1.440]), and aspartate aminotransferase-to-platelet ratio index (APRI) (P = 0.005, HR [1.911, 1.221–2.989]) were independent risk factors for recurrence.

**Conclusion:**

Patients receiving SBRT for HBV-related sHCC may be at greater risk of recurrence if they have a high APRI score combined with advanced age and large tumor size.

## 1 Introduction

Primary liver cancer is the sixth most common cancer in the world and the third leading cause of cancer death (1). Hepatocellular carcinoma (HCC) is the dominant type of primary liver cancer, and hepatitis B virus (HBV) infection plays a crucial role in the development of HCC in Asia. In the past, radiation therapy was commonly used to treat advanced HCC. For the treatment of small hepatocellular carcinoma (sHCC), liver resection has been recommended as the first-line treatment according to the European Association for the Study of the Liver (EASL) and the American Association for the Study of Liver Diseases (AASLD) (2, 3). Radiofrequency ablation (RFA) can serve as an alternative option for sHCC patients who are ineligible for surgery because of cirrhosis-related portal hypertension. However, the location of the tumor (e.g., close to large vessels and the bile duct) may increase the risk of complications or may result in failure to completely eradicate the tumor with RFA. Advances in radiation therapy, particularly stereotactic body radiation therapy (SBRT), have allowed the enhanced delivery of large ablative doses of radiotherapy to the tumor while sparing surrounding critical tissue. SBRT is increasingly recognized as a valuable treatment for sHCC, and the survival benefits of SBRT in the treatment of sHCC can be similar to those of liver resection or RFA (4–8). Previous studies demonstrated that SBRT might be beneficial for HBV-related sHCC patients who are unfit for surgery and provide a comparable prognosis to surgery (4). Some studies have shown that SBRT achieves a therapeutic effect similar to that of RFA. SBRT is recommended as a category 2B treatment for patients with HCC (9). Unfortunately, understanding of the risk factors for recurrence in patients with sHCC after SBRT remains limited. Researchers have evaluated recurrence as well as risk factors for the recurrence of sHCC after SBRT treatment, but the results may be influenced by the number of tumors (not a single lesion), the treatment received (such as surgery), the different causes of sHCC (such as HCV), and the size of the tumor (>5 cm) (10–12). This study aimed to identify risk factors for recurrence in patients with HBV-related sHCC after SBRT.

## 2 Materials and Methods

### 2.1 Study Population

**Figure 1:**
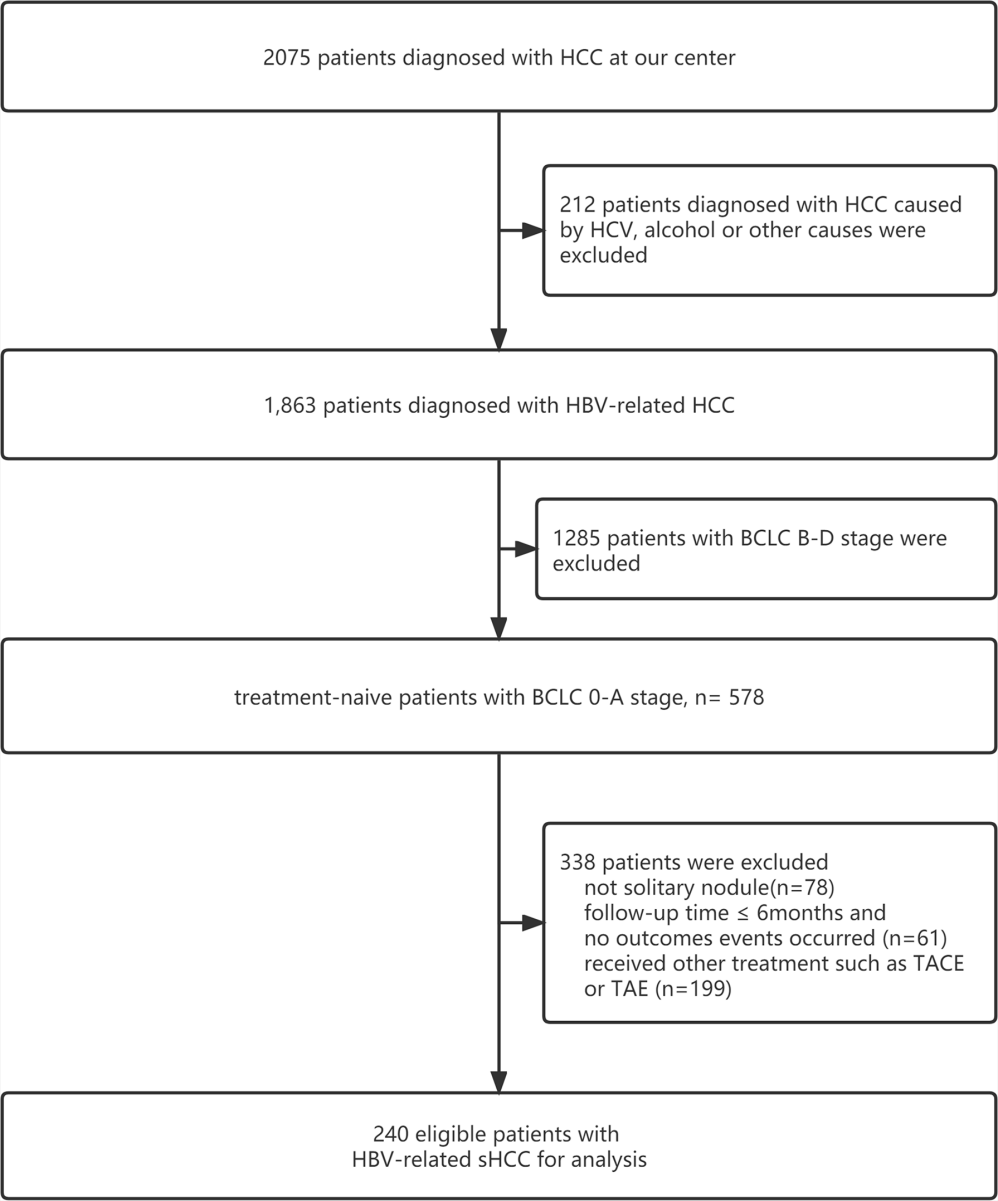
Flowchart of patients and study design.

This study included 240 patients with HBV-related sHCC admitted to the Fifth Medical Center of PLA General Hospital between March 2011 and March 2020. The inclusion criteria were as follows: (1) a diagnosis of HCC based on the pathology of liver biopsy, image examination, or biomarkers of HCC such as alpha-fetoprotein (AFP); (2) a single nodule ≤5 cm and without vascular infiltration or lymph node or extrahepatic metastases (13); (3)no history of transhepatic arterial chemotherapy and embolization (TACE), RFA, or other therapies before receiving SBRT; (4) with HBV infection (treated with entecavir or tenofovir) and no other causes of HCC, such as HCV infection or aflatoxin; (5) classified as Child–Pugh Class A or B (CP-A or CP-B) and an Eastern Cooperative Oncology Group performance status (ECOG PS) score of 0 to 1; (6) residual liver volume of at least 700 ccs and distances between the lesion and normal organs (stomach, duodenum, colon, and bowel) greater than 3 mm (patients whose tumors were close to the stomach and/or duodenum underwent endoscopy examination to exclude ulcers); (7) diagnosis of with liver cirrhosis or noncirrhosis based on clinical manifestations, image examination (ultrasound, transient elastography, CT, or MRI), esophageal gastric varices in gastroscopy, thrombocytopenia, etc.; and (8) diagnosis of portal hypertension (PHT) or non-PHT by presence of significant splenomegaly, imaging findings of umbilical vein recanalization and/or portal system shunt, and a preoperative platelet count <100 (14, 15). The flowchart is shown in **Figure 1**.

A standardized data form was created to collect all the relevant clinical information. The form contained the following sections: (1) demographic and clinical characteristics of the patients (including age, sex, cirrhosis, family history of HCC, duration of HBV infection, and time and plan for HBV treatment); (2) laboratory test results, including HBV surface antigen (HBsAg), HBV-DNA, alpha-fetoprotein (AFP), platelets (normal range of 100–300 ∗ 10^9^/L), alanine aminotransferase (ALT, the normal range of 9–50 IU/L), aspartate aminotransferase (AST, the normal range of 15–40 IU/L), albumin (ALB, the normal range of 40–55g/L), total bilirubin (TBIL, the normal range of 2–20 umol/L), albumin-bilirubin (ALBI) grade, Child–Pugh class, and APRI (<2 or ≥2) (16), which was calculated using the following formula: [AST(IU/L)/upper limit normal]/PLT(×10^9^/L) × 100; (3) tumor characteristics, including tumor size, location, and Barcelona Clinic Liver Cancer (BCLC) stage 0-A; and (4) total dose of SBRT and follow up. This retrospective cohort study was approved by the Institutional Review Board of Beijing 302 Hospital and was conducted in accordance with the Declaration of Helsinki and internationally accepted ethical guidelines.

### 2.2 Radiation Treatment Planning

All the enrolled patients were implanted with 4 to 6 fiducial markers one week before SBRT treatment (CyberKnife, Accuray, USA). The distance between the markers and the lesion was less than 6 cm. The radiation oncologists contoured the gross tumor volume (GTV), planning target volume (PTV), and organs at risk (normal liver, kidneys, esophagus, stomach, duodenum, bowel, gallbladder, common bile duct, and spinal cord). GTV was defined as a visible lesion on imaging examination. The PTV was expanded to 3–5 mm around the GTV, and it contoured 100% of the GTV and avoided the organs at risk. All the plans were designed using G4 CyberKnife Multiplan (Version 4.0.2) and VSI CyberKnife MultiPlan (Version 4.6.1). Normal tissue tolerance doses complied with the AAPM TG-101 report (17). A total dose of 48–54 Gy was delivered over 5–8 fractions. The biological effective doses (BED_10_) were 83.3 to 102.6 Gy. Dynamic respiration tracking and fiducial tracking were simultaneously used during the treatment. The normal liver volume (NLV) was equal to the total liver volume minus the GTV, and the NLV was required to be greater than or equal to 800 cm^3^.

### 2.3 Follow-Up

Follow-up visits were performed every 3–4 months, and laboratory tests, including complete blood count (CBC), liver function tests, coagulation function, AFP, and imaging (CT or MRI) of the liver and other organs, such as the lungs and bones, were performed. The endpoint was the first recurrence of HCC, which was defined as an intrahepatic recurrence, metastasis, or any progression of HCC after receiving SBRT. Local recurrence was calculated from the date of SBRT to the date of treated-lesion recurrence or progression. Intrahepatic recurrence was calculated from the date of SBRT to the date of intrahepatic recurrence or progression. Based on the imaging examination and HCC biomarkers, a group composed of clinicians and radiologists assessed the recurrence of HCC. The imaging examination was evaluated independently by two faculty radiologists with more than six years of experience. Disagreements were resolved by a third radiologist with over 10 years of experience.

Tumor response was evaluated as described in the Response Evaluation Criteria in Solid Tumors (RECIST) version 1.1 (18) and Modified Response Evaluation Criteria in Solid Tumors (mRECIST) (19) after the completion of SBRT.

### 2.4 Statistical Analysis

By using the Shapiro test, we determined whether values were within a normal distribution, and the results are presented as the mean ± standard deviation or median (interquartile ranges [IQRs]). We presented the recurrence rate as a ratio (95% confidence interval [95% CI]). Actuarial recurrence rates were determined, and survival curves were constructed based on the Kaplan–Meier method. These data were compared with the log-rank test. To identify potential prognostic factors for recurrence, univariate and multivariate Cox proportional hazards regression model analyses were performed. R software was used for statistical analyses with the “survival” and “survminer” packages (http://www.r-project.org). A P-value of <0.05 was considered statistically significant.

## 3 Results

### 3.1 Patient Characteristics

The clinical characteristics of 240 patients with HBV-related sHCC are summarized in **Table 1**. The average age of the patients was 54 years, 180 patients were men, and 227 patients had cirrhosis. Most patients had Child–Pugh A (91.3%) and BCLC Stage A (93.7%). Almost all the patients (99.2%) tested positive for HBsAg. A total of 40.4% of patients had received antiviral treatment.

**Table 1:** Baseline characteristics of the patients.

Variable	Total (n = 240)	Non-recurrence group (n = 106)	Recurrence group (n = 134)	P
Age	54.0 ± 9.4	52.4 ± 9.0	55.4 ± 9.4	0.014
Sex (n,%)				0.653
Male	180 (75.0)	78 (73.6)	102 (76.1)	
Female	60 (5.0)	28 (26.4)	32 (23.9)	
Tumor diameter (cm)	2.10 [1.50, 3.03]	1.95 [1.43, 2.78]	2.20 [1.60, 3.18]	0.029
Child–Pugh (n, %)				0.132
A	219 (91.3)	100 (94.3)	119 (88.8)	
B	21 (8.7)	6 (5.7)	15 (11.1)	
AFP (ng/ml)	14.7 [4.3,118.8]	9.7 [4.1, 116.8]	19.4 [4.7, 119.0]	0.407
Liver cirrhosis (n, %)				0.470
Yes	227	99 (93.4)	128 (95.5)	
No	13	7 (6.6)	6 (4.5)	
PHT (n,%)				0.327
Yes	186 (77.5)	79 (74.5)	107 (79.9)	
No	54 (22.5)	27 (25.5)	27 (20.1)	
ALBI grade (n, %)				0.649
1	124 (51.7)	56 (52.8)	68 (50.7)	
2	106 (44.2)	47 (43.5)	59 (44.0)	
3	3 (1.3)	3 (2.8)	7 (5.2)	
HBsAg status (n, %)				1.000
Yes	238 (99.2)	105 (99.1)	133 (99.3)	
No	2 (0.8)	1 (0.9)	1 (0.7)	
HBV-DNA (n, %)				0.425
Positive	76 (31.7)	35 (33.0)	41 (30.6)	
Negative	153 (63.8)	62 (58.5)	91 (67.9)	
Loss	11 (4.6)	9 (8.5)	2 (1.5)	
Platelet (10^9^/L)	114.5 [74.0, 161.0]	124.5 [82.8, 163.3]	105.5 [63.8, 158.0]	0.068
Albumin (g/L)	39.0 [36.0, 42.0]	40.0 [36.0, 42.0]	39.0 [35.3, 42.0]	0.490
ALT (IU/ml)	27.0 [19.0, 38.0]	25.0 [19.0, 38.0]	27.5 [19.0, 38.0]	0.987
AST (IU/ml)	30.0 [23.0, 40.0]	29.0 [22.3, 36.0]	30.5 [23.0, 42.0]	0.114
TBIL (umol/L)	14.75 [11.35, 21.32]	14.50 [11.47, 22.07]	15.2 [11.28, 20.98]	0.659
Family History (n, %)				0.062
Yes	210 (87.5)	88 (83.0)	122 (91.0)	
No	30 (12.5)	18 (17.0)	12 (9.0)	
Alcohol (n, %)				0.308
Yes	104 (43.3)	50 (47.2)	54 (40.3)	
No	136 (56.7)	56 (52.8)	80 (59.7)	
PVT (n,%)				0.479
Yes	6 (2.5)	4 (3.8)	2 (1.5)	
No	234 (97.5)	102 (96.2)	132 (98.5)	
Antivirus therapy (n, %)				0.170
Yes	97 (40.4)	56 (52.8)	41 (30.6)	
No	127 (52.9)	38 (35.8)	8,966.4)	
no data	16 (6.7)	12 (11.3)	4 (3.0)	
Total dose of SBRT	54.0 [49.0, 54.0]	54.0 [49.0, 54.0]	50.0 [49.0, 54.0]	0.156
ECOG				0.191
0	190 (79.2)	88 (83.0)	102 (76.1)	
1	50 (20.8)	18 (17.0)	32 (23.9)	

Values presented as mean ± standard deviation or median (quartile,[Q1, Q3]).

BCLC, Barcelona Clinic Liver Cancer; AFP, alpha-fetoprotein; PHT, portal hypertension; ALBI, albumin-bilirubin; HBsAg, hepatitis B virus surface antigen; ALBI, albumin-bilirubin; ALT, alanine aminotransferase; AST, alanine aminotransferase; TBIL, total bilirubin; PVT, portal vein thrombosis; SBRT, stereotactic body radiation therapy; ECOG, Eastern Cooperative Oncology Group.

### 3.2 Tumor Response and Local Control

**Figure 2:**
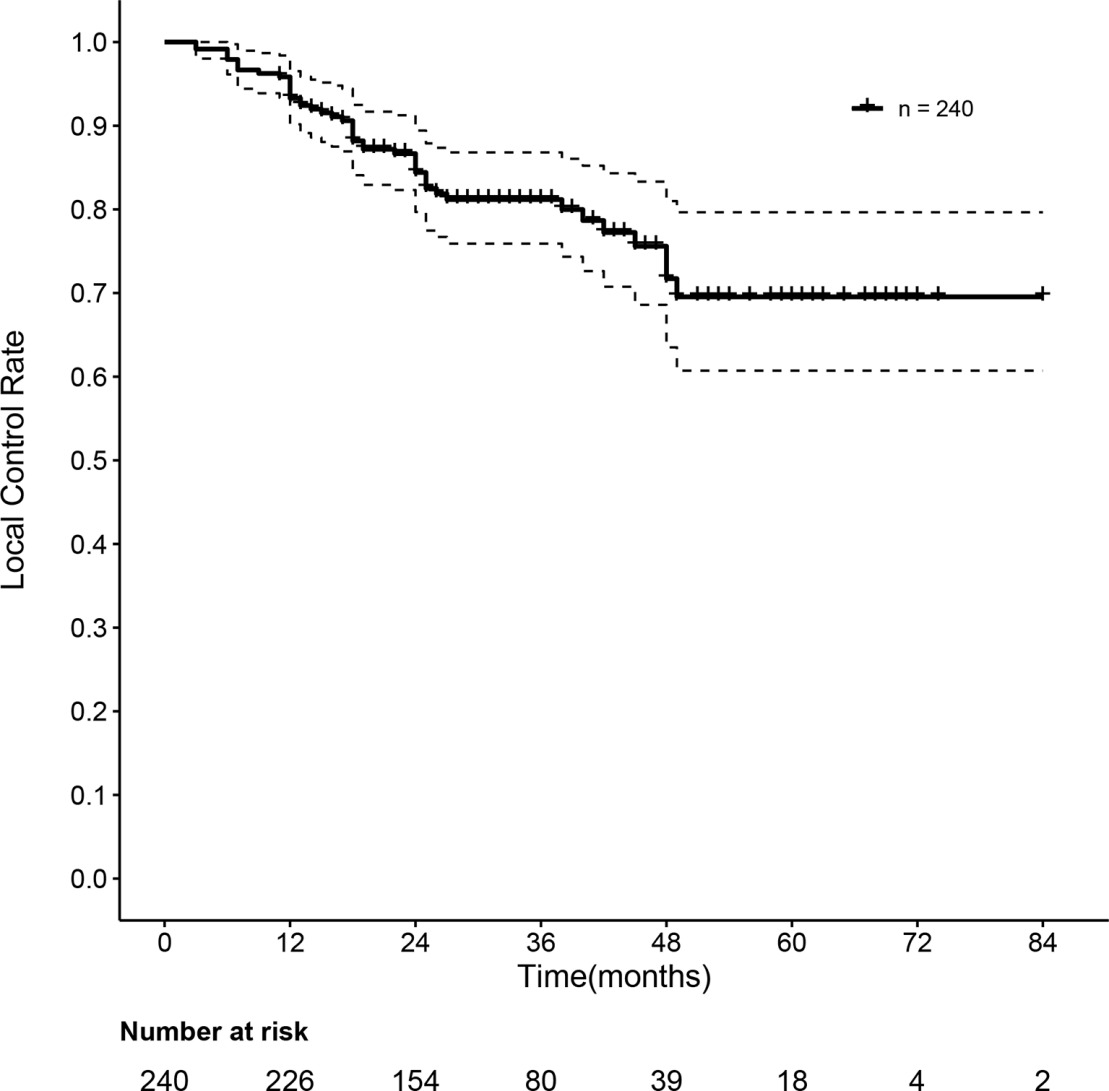
Curve of the local control rate of 240 HBV-related sHCC patients after SBRT. HBV, hepatitis B virus; sHCC, small hepatocellular carcinoma; SBRT, stereotactic body radiation therapy.

As summarized in **Table 2**, when we evaluated the tumor response according to RECIST 1.1, the overall response was observed in 223 of 240 patients (92.9%) after three months of SBRT. A total of 114 (47.5%) and 109 (45.4%) patients achieved complete response (CR) and partial response (PR), respectively. Stable disease (SD) was observed in 11 patients (4.6%), and progressive disease (PD) was observed in 6 patients (2.5%). However, when we evaluated the tumor response according to mRECIST, an overall response was observed in 236 of 240 patients (93.3%) after three months of SBRT. A total of 152 (63.4%) and 84 (35.0%) patients achieved complete response (CR) and partial response (PR), respectively. Stable disease (SD) was observed in only 2 patients (0.8%), and progressive disease (PD) was also observed at the same rate as SD. The one- and two-year local control rates were 93.3% (95% CI: 90.2–96.5%) and 84.4% (95% CI: 79.7–89.4%), respectively. The median local control time was 31 months. The curve of the local control rate is shown in **Figure 2**.

**Table 2:** Tumor Response at three months after SBRT.

Tumor response	RECIST 1.1	mRECIST
	N (%)	N (%)
Overall response rates*	223 (92.9)	236 (98.3)
Complete response	114 (47.5)	152 (63.3)
Partial response	109 (45.4)	84 (35.0)
Stable Disease	11 (4.6)	2 (0.8)
Progressive disease	6 (2.5)	2 (0.8)

RECIST, Response Evaluation Criteria in Solid Tumors; mRECIST, modified Response Evaluation Criteria in Solid Tumors; SBRT, Stereotactic Body Radiation Therapy.

*Defined as the proportion of lesions that had complete or partial responses.

### 3.3 Follow-Up and Recurrence

**Figure 3:**
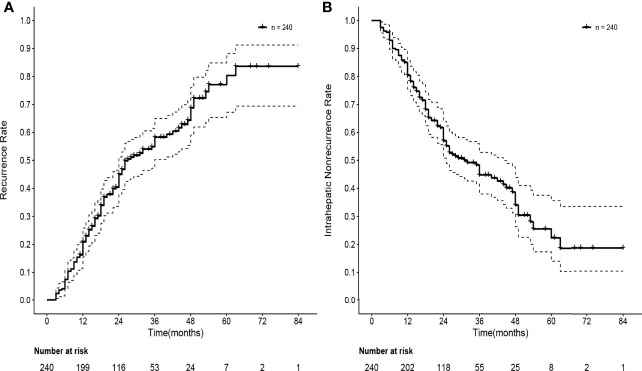
Curves of recurrence rate and intrahepatic nonrecurrence rate of 240 HBV-related sHCC patients after SBRT. **(A)** Curve of recurrence rate of 240 HBV-related sHCC patients. **(B)** Curve of intrahepatic nonrecurrence of 240 HBV-related sHCC patients. HBV, hepatitis B virus; sHCC, small hepatocellular carcinoma; SBRT, stereotactic body radiation therapy.

The median follow-up time was 30 months. After SBRT, 134 patients experienced HCC recurrence, and the median time to recurrence was 27 months. The actuarial rates of overall recurrence were 20.9% (95% CI: 15.6–25.9%) and 45.0% (95% CI: 37.9–51.2%) at one and two years, respectively. A total of 127 patients experienced intrahepatic recurrence and 7 patients experienced extrahepatic metastasis. The one- and two-year intrahepatic recurrence rates were 19.6% (95% CI: 14.4–24.5%) and 43.4% (95% CI: 36.4–49.6%), respectively. The median time to intrahepatic recurrence was 31 months. Forty-six patients experienced local recurrence. The local recurrence rates were 6.7% (95% CI: 3.5–9.8%) and 15.6% (95% CI: 10.6–20.3%) at one and two years, respectively. The median time to local recurrence was 31 months. The overall recurrence rate and intrahepatic nonrecurrence rate curves are shown in **Figure 3**.

### 3.4 Univariate and Multivariate Analysis of Risk Factors for Recurrence

According to univariate Cox regression analysis, age, tumor size, and APRI score were all correlated with recurrence, and we chose the variables for multivariate analysis based on the P <0.05 cutoff value. In both univariate and multivariate Cox regression analyses, older age (p = 0.029, HR = 1.019 [1.002–1.037]), larger tumor size (P = 0.012, HR = 1.227 [1.045–1.440]) and APRI ≥2 (P = 0.005, HR = 1.911 [1.221–2.989]) were all associated with recurrence in patients with HBV-related sHCC after SBRT (**Table 3**).

**Table 3:** Risk factors for Recurrence: Univariate and multivariate Cox regression analyses.

Variable	Univariate analysis			Multivariate analysis		
	HR	95% CI	P	HR	95% CI	P
Age (years)	1.019	1.002–1.035	0.026	1.018	1.001–1.035	0.038
Sex	0.898	0.603–1.337	0.596			
Liver cirrhosis (yes)	1.363	0.600–3.095	0.459			
Family history of HCC (yes)	0.658	0.363–1.190	0.166			
Tumor size (cm)	1.227	1.049–1.435	0.011	1.182	1.005–1.390	0.043
PHT (yes)	1.264	0.828–1.930	0.279			
ALBI grade	1.129	0.841–1.514	0.421			
HBV-DNA status (positive)	0.993	0.687–1.437	0.972			
Antivirus therapy (Yes)	1.158	0.799–1.678	0.440			
AFP (ng/ml)	1.000	1.000–1.000	0.162			
PLT (10^9^/L)	0.999	0.996–1.002	0.622			
ALT (IU/L)	0.999	0.993–1.004	0.708			
AST (IU/L)	1.004	1.000–1.007	0.069			
APRI (≥2)	2.073	1.318–3.261	0.002	1.969	1.247–3.111	0.004
ALB (g/L)	0.976	0.946–1.007	0.129			
Scr (umol/L)	1.004	0.996–1.002	0.415			
Total dose of SBRT (Gy)	0.981	0.945–1.017	0.298			
Child–Pugh (B)	1.650	0.961–2.831	0.069			

HR, hazard ratio; 95% CI, 95% confidence interval; PHT, portal hypertension; ALBI, albumin-bilirubin; AFP, albumin-bilirubin; PLT, platelet; ALT, alanine aminotransferase; AST, alanine aminotransferase; APRI, aspartate aminotransferase-to-platelet ratio index; ALB, albumin; Scr, serum creatinine; SBRT, stereotactic body radiation therapy.

### 3.5 Recurrence of Patients With Different APRI Scores

**Figure 4:**
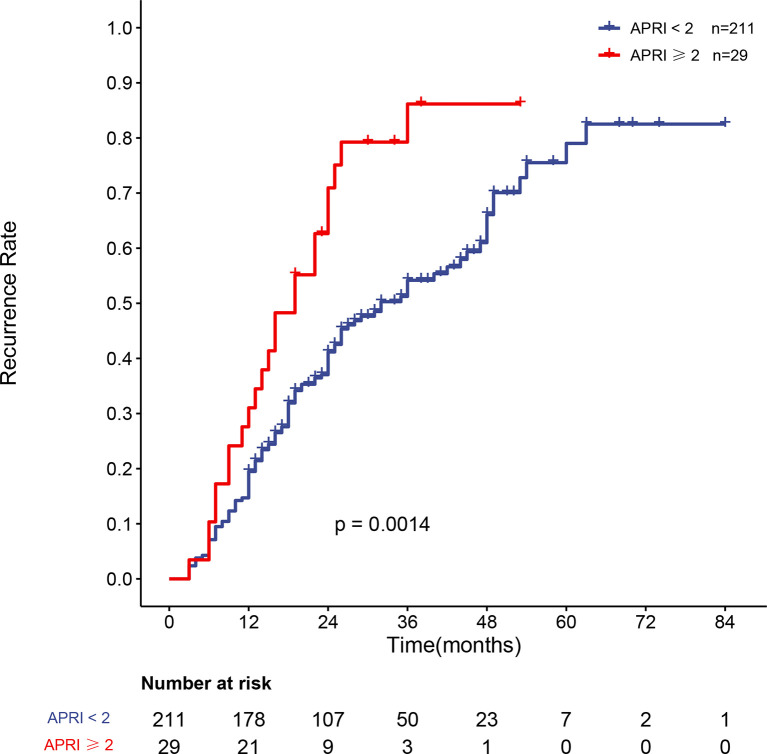
Curve of the recurrence rate of 240 HBV-related sHCC patients with an APRI <2 and an APRI ≥2. HBV, hepatitis B virus; sHCC, small hepatocellular carcinoma; APRI, aspartate aminotransferase-to-platelet ratio index.

**Figure 5:**
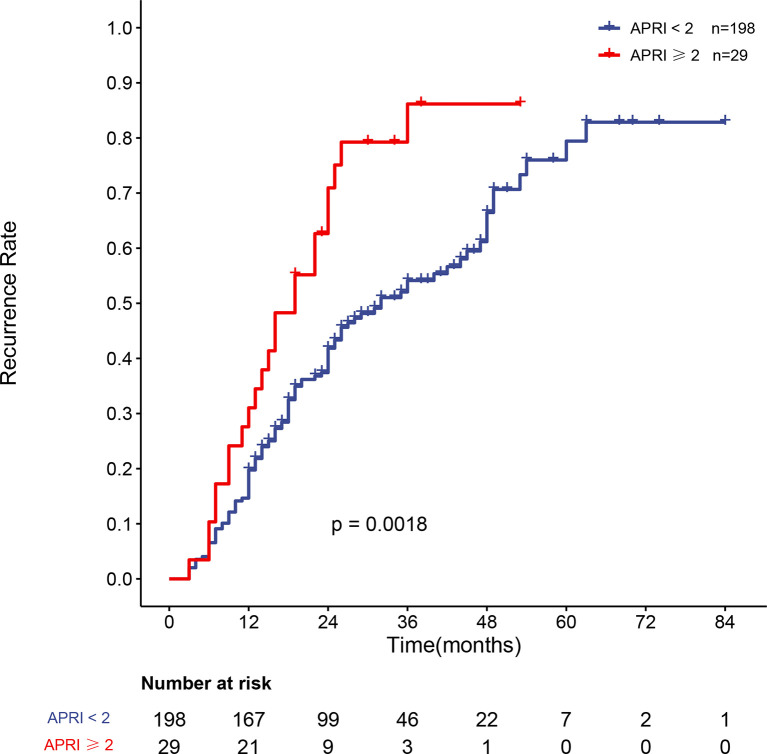
Curve of the recurrence rate of 227 HBV-related sHCC patients with cirrhosis with an APRI <2 and an APRI ≥2. HBV, hepatitis B virus; sHCC, small hepatocellular carcinoma; APRI, aspartate aminotransferase-to-platelet ratio index.

APRI was an independent risk factor for recurrence in HBV-related sHCC patients after SBRT. The overall recurrence rates between the two groups differed significantly; the one- and two-year recurrence rates were 19.5% (95% CI: 13.9–24.7%) and 41.2% (95% CI: 33.7–47.7%), respectively, in patients with an APRI <2, while the one- and two-year recurrence rates were 31.0% (95% CI: 12.0–46.0%) and 29.1% (95% CI: 47.5–83.9%), respectively, in patients with an APRI ≥2 (P = 0.0014). **Figure 4** shows the recurrence rate curve for patients with different APRI scores. Due to the high rate of cirrhosis in the recurrence and nonrecurrence groups of our study (all over 90%), we plotted the recurrence curve for patients diagnosed with cirrhosis (n = 227) with different APRI scores (**Figure 5**). There was also a significant difference in the recurrence rates between the two groups. The one- and two-year recurrence rates were 19.8% (95% CI: 14.0–25.1%) and 41.9% (95% CI: 34.1–48.7%), respectively, in patients with an APRI <2, while the one- and two-year recurrence rates were 31.0% (95% CI: 12.0–46.0%) and 29.1% (95% CI: 47.5–83.9%), respectively, in patients with an APRI ≥2 (P = 0.0018).

## 4 Discussion

In this study of patients with sHCC, SBRT was associated with a high tumor response rate (92.9% by RECIST 1.1 and 98.3% by mRECIST at three months) and local control rate (93.3 and 84.3% at one and two years). These results show the clinical benefits of using SBRT for treating sHCC and are similar to those reported in a previous study (20). Our analysis demonstrates that older age, larger tumor diameter, and a higher APRI score are risk factors for recurrence.

Multiple retrospective studies have explored the prognosis and risk factors for recurrence in HCC patients after SBRT. However, the inclusion criteria in these studies varied, with some trials including patients with portal vein tumor thrombus (11) and others including patients who had experienced a relapse or received other prior treatments (12, 21–23). Moreover, patients with various tumor sizes and numbers were included (8, 22). There are multiple causes of HCC (8, 23). Thus, it was difficult to draw a consistent conclusion regarding the risk factors for recurrence among sHCC patients who were treated with SBRT. Therefore, we designed the current trial to include patients with HBV-related sHCC with no history of treatment before SBRT.

Age is a well-known risk factor for HCC recurrence and was a significant risk factor in our study. As age increases, the rate of the tumor spread and recurrence increases. Previous studies have shown that tumor size is a risk factor and predictor of sHCC recurrence and prognosis in sHCC patients treated with SBRT. However, there is no specific cutoff value for tumor size (11, 24). It is worth noting that in this study, larger tumor size was also a risk factor for recurrence, but when we divided patients into <2 cm and 2–5 cm groups based on tumor size and assessed the relationship of tumor size with tumor recurrence, the Cox univariate analysis showed significant differences between the recurrence and nonrecurrence groups, but the multivariate analysis did not. Accordingly, larger tumor size has also been associated with a poor prognosis in previous studies, but the specific tumor size threshold still needs further exploration and analysis.

This study supports the findings of other studies that indicate that the APRI is independently associated with poor survival among HCC patients (25, 26). Hung et al. reported that the APRI was a reliable marker for assessing and predicting HCC recurrence rates (25). Shen et al. showed that the APRI was associated with the prognosis of patients with solitary HBV-related sHCC (26). The results of previous studies were predominantly based on patients with HBV-related sHCC who were treated with liver resection. Our study suggests that the APRI is also an independent predictor of recurrence risk among HBV-related sHCC patients who received SBRT. The analysis of the recurrence rate revealed a significant difference in the risk of recurrence between patients with an APRI <2 and those with an APRI ≥2. Obtaining patient APRI scores is inexpensive and easy, requiring only routine biochemical tests performed before treatment without additional blood draws. A higher APRI score suggests the existence of liver fibrosis and liver cirrhosis (16, 27) in chronic hepatitis B patients and is associated with liver cirrhosis in patients with hepatocellular carcinoma (28). Liver cirrhosis has been considered as a predictor of the occurrence and recurrence of sHCC (29). However, cirrhosis was not a significant risk factor in univariable and multivariable analyses in our study. This finding may be related to the high proportion of cirrhosis in both our recurrence (95.5%) and nonrecurrence (93.4%) groups. Therefore, we selected patients who were enrolled in this study and had cirrhosis, and we performed a survival analysis of the recurrence rates between the groups with different APRI scores. The results still suggested a significant difference in recurrence between patients with an APRI <2 and those with an APRI ≥2. This phenomenon may indicate that even in patients with liver cirrhosis, different APRI scores reflect different degrees of cirrhosis. An elevated APRI would require the serum AST level to be increased, with a relatively lower platelet count. There are many reasons for increases in the AST level, such as damage due to cirrhosis and the release of AST from injured mitochondria with impaired clearance of HBV. In our study, the median AST level was higher in our recurrence group, reflecting a greater degree of underlying liver cirrhosis, liver regeneration, and restoration of function after SBRT. Another unique characteristic of the APRI is that this score includes the platelet count. In our study, the level of platelets was lower and the number of patients diagnosed with PHT was higher in the recurrence group than in the nonrecurrence group. Reduced platelet counts may be a result of liver cirrhosis and portal hypertension. Platelet count was identified as a prognostic factor for predicting HCC recurrence after resection and the existence of microvascular invasion, which was associated with HCC recurrence in previous studies (30, 31). However, AST level, platelet count, and other well-known risk factors for predicting prognosis, such as Child–Pugh grade, were not significant risk factors for recurrence in our analysis because most of the enrolled patients were diagnosed with BCLC A and Child–Pugh A stage tumors. As a result, there were no apparent differences between these factors in our analysis. Thus, we need to study additional sHCC patients who were treated with SBRT to explore more effective indicators such as the APRI instead of simply using the platelet count or AST level to predict the recurrence.

In addition to age, tumor size, and APRI score, more clinical studies with larger sample sizes may be needed to further explore factors that influence the recurrence of HBV-related sHCC. Previous studies have shown that a higher BED_10_ (≥100 Gy) may improve prognosis and reduce recurrence in patients with HCC after SBRT (32, 33). A higher BED_10_ may improve local control and therapeutic efficacy, but it can also cause an increased risk of liver damage after treatment. Since most patients included in this study had a history of cirrhosis and were prone to adverse reactions, such as radiation-induced liver damage, the BED_10_ range of patients enrolled in this study was 83.3–102.6 Gy. More studies may be needed to explore more appropriate doses of BED to achieve a balance between therapeutic efficacy and the occurrence of adverse effects.

The advantage of our study is that we included more participants than most previous similar studies, and all the selected patients were screened with more stringent admission criteria (HBV-related sHCC (single lesion ≤5 cm) without any prior treatment before SBRT). Additionally, the study revealed that the APRI, as a feasible and noninvasive indicator, could also determine the recurrence risk of patients with HBV-related sHCC after SBRT treatment and determine the population of HBV-related sHCC patients who are likely to benefit from SBRT. Of course, our study also has certain limitations because it was a single-center retrospective study, and the results of this study are mainly based on the Chinese population.

## 5 Conclusion

HBV-related sHCC patients who were treated with SBRT may have a higher risk of recurrence if they have a high APRI score, advanced age, and large tumor size.

## Data Availability Statement

The original contributions presented in the study are included in the article/supplementary material. Further inquiries can be directed to the corresponding authors.

## Ethics Statement

Written informed consent was obtained from the individual(s) for the publication of any potentially identifiable images or data included in this article.

## Author Contributions

YH and JL collected the data, finished the manuscript, and prepared the figures and tables. XX and WL gave constructive guidance. JP, HC, NT, QK, and YY participated in the design of this article. All authors listed have made a substantial, direct, and intellectual contribution to the work and approved it for publication.

## Funding

This study was supported by the National Science and Technology Major for Infectious Diseases (Nos. 2017ZX10302201-004-009 and 2017ZX10203202-003), the Beijing Municipal Science and Technology Commission of Major Projects (No. D171100003117005, D161100002716002, and D161100002716003), and the National Science and Technology Major Special Project for New Drug Development (No. 2018ZX09201016).

## Conflict of Interest

The authors declare that the research was conducted in the absence of any commercial or financial relationships that could be construed as a potential conflict of interest.

## Publisher’s Note

All claims expressed in this article are solely those of the authors and do not necessarily represent those of their affiliated organizations, or those of the publisher, the editors and the reviewers. Any product that may be evaluated in this article, or claim that may be made by its manufacturer, is not guaranteed or endorsed by the publisher.
